# Normal Ovariotomy, Another Case

**Published:** 1874-03

**Authors:** 


### Editorial.

---

We feel assured that the judgment of our readers will approve our decision in giving up so large a portion of our space in the current number of the Journal, to the history of the Siamese Twins, and the autopsy, and discussion by the Philadelphia College of Physicians; for all of which we are indebted to the Philadelphia *Medical Times.*

We learn, through a private correspondent in New York city, that the new operation of *Normal Ovariotomy,* has been repeated in that city, in November last, and that "on this day (17th February) the patient, a young lady, was discharged perfectly cured, and has gone home to be married." We shall doubtless have the full report of the case through one of the Medical Journals in due time.

